# Prevalence of *mecA* gene among *staphylococci* from clinical samples of a tertiary hospital in Benin City, Nigeria

**DOI:** 10.4314/ahs.v17i4.7

**Published:** 2017-12

**Authors:** Ephraim Ehidiamen Ibadin, Idahosa Onaiwu Enabulele, Fowora Muinah

**Affiliations:** 1 Medical Microbiology Unit, Medical Laboratory Services, University of Benin Teaching Hospital, Benin City, Nigeria; 2 Department of Microbiology, Faculty of Life Sciences, University of Benin, Benin City, Nigeria; 3 Molecular Biology and Biotechnology Division, Nigerian Institute of Medical Research, Yaba, Nigeria

**Keywords:** *Staphylococcus aureus* (*S. aureus*), coagulase-negative *staphylococci* (CoNS), *mecA* gene, methicillin-resistance

## Abstract

**Background:**

The *staphylococci* have increasingly been associated with infections worldwide and anti-microbial resistance has made these versatile pathogens more recalcitrant in the hospital setting.

**Objectives:**

This study sought to investigate the occurrence and distribution of *Staphylococcus* species as well as determine the prevalence of methicillin resistant *Staphylococcus aureus* (MRSA) and methicillin resistant coagulase negative *staphylococci* (MRCoNS) among clinical samples from University of Benin Teaching Hospital (UBTH) in Benin City.

**Methods:**

Ninety one (91) clinical isolates comprising *S. aureus* and Coagulase Negative *staphylococci* (CoNS) were recovered from routine clinical specimens and anti-microbial susceptibility tests were carried out. Polymerase Chain Reaction (PCR) was thereafter carried out on these isolates to detect *mecA* gene.

**Results:**

*Staphylococcus* species had its highest prevalence from infected wounds of patients (28.8%) while urine samples showed the least (5.4%). The highest level of resistance was to ceftazidime (*S. aureus* - 68%, CoNS - 75.6%) while the least resistance was observed for meropenem (*S. aureus*- 26%, CoNS- 46.3%). Using phenotypic method (with 1µg oxacillin antibiotic disc), the distribution of MRSA and MRCoNS was 44.0% and 46.3% respectively. PCR analysis showed that 38.0% of *S. aureus* and 41.5% of the CoNS had *mecA* gene respectively; wound swabs showed the highest prevalence with 30.5% of *staphylococcal* isolates being mecA gene positive. There was also no significant association between the *Staphylococcal* isolates and their isolation rate, isolation site and *mecA* gene distribution (p > 0.05).

**Conclusion:**

This study draws attention on the increase in the prevalence of *mecA* gene (39.6%) and an increase in multidrug resistant *staphylococci* when compared to previous studies in our country; it recommends laboratory guidance and periodic review to stem the tide of resistance.

## Introduction

*Staphylococcus aureus* is a non-motile, aerobic or facultative anaerobic Gram positive *coccus* that inhabits the nasal membranes and skin of warm blooded animals and man, in whom it could cause a range of infections from mild, such as skin infections and food poisoning, to life threatening, such as pneumonia, sepsis, osteomyelitis and infectious endocarditis^1^. The CoNS are part of the normal flora of human skin^2^, these organisms have relatively low virulence but are increasingly recognized as agents of clinically significant infection of the bloodstream and other sites^3^. Risk factors for CoNS infection include foreign bodies (such as indwelling prosthetic devices or intravascular catheters) and immune compromise^3^.

The introduction of methicillin in 1960 as an alternative antibiotic for the treatment of beta-lactamase (penicillinase) producing *S. aureus* was greeted with resistance almost immediately^4^,^5^. Since that time, strains of *S. aureus* resistant to methicillin emerged and became a major clinical problem within hospitals in Europe in the 1960s^6^, in the United States in the 1970s^7^, and in several countries of the world^8^. Resistance to methicillin and semisynthetic penicillins has been observed in more than 80% of coagulase-negative *Staphylococcal* isolates^9^. MRSAs and MRCoNS have therefore emerged as the most important cause of hospital-acquired infections (HAI) and community-acquired infections (CAI), resulting in increased morbidity and mortality in the hospital settings^10^,^11^.

The specific genetic mechanism of its resistance in MRSAs has been identified as a mobile genetic element (*staphylococcal* cassette chromosome mec) integrated into the *S. aureus* chromosome, within which the mecA gene encodes a specific methicillin-resistant transpeptidase known as penicillin-binding protein 2a (PBP2a)^12^. This protein has a low affinity for beta-lactam antimicrobial drugs, thus bacteria expressing this protein are resistant to all types of these drugs^13^, and in recent times; aminoglycosides and quinolones^13^.

In Nigeria, the occurrence of MRSA was first documented by researchers in 1987 with its prevalence pegged at 50.6%^14^. Subsequent studies have seen a variation in its prevalence^15^,^16^,^17^. Literature is growing on CoNS and its incrimination in clinical infections in Nigeria^18^,^19^; several factors affect the reliability of the disk diffusion technique for the detection of methicilin resistance among the *staphylococci*^17^,^20^. Though molecular methods have emerged as the gold standard, there is still little data on the distribution of MRSA and MRCoNS in our locality.

This study therefore aims at evaluating the prevalence and distribution of MRSAs and MRCoNS among *staphylococci* isolated from clinical samples by determining the distribution of *mecA* gene among these organisms in Benin City using PCR based method.

## Materials and methods

### Sources of clinical specimens

The *staphylococci* isolates used in this study were obtained by serial sampling. Ninety one (91) consecutive non-repetitive clinical isolates comprising *S. aureus* and CoNS were recovered from routine clinical specimens sent to the diagnostic laboratory of Medical Microbiology department, University of Benin Teaching Hospital, Benin city (UBTH).The clinical samples were; wound swabs (25), endocervical swabs (4), high vaginal swabs (3), eye swabs (4), ear swabs (9), urethral swabs (3), seminal fluid (10), blood (17), urine (12), aspirates (2) and urinary catheter tips (2). These specimens were collected from patients admitted in wards (in-patients), and from patients attending out-patient clinics.

### Isolation and identification

The isolates were identified to be *S. aureus* following Gram stain that showed Gram positive *cocci* and biochemical tests which showed positive results for catalase and coagulase (slide and/or tube) as described in standard Medical Microbiology laboratory manual^21^. Similarly isolates identified as CoNS were Gram positive *cocci* and catalase positive but were negative for both slide and tube coagulase tests. The isolates were thereafter stored at 4°C on Mueller Hinton agar slants for further work.

### Antimicrobial susceptibility testing

In vitro anti-bacterial susceptibility tests were performed on each isolate using anti-bacterial drugs. Plates were prepared with Mueller Hinton's agar for use in the Kirby-Bauer method. Broth cultures were adjusted to turbidity standard, equivalent to McFarland 0.5. This was then used for disc diffusion according to Clinical and Laboratory Standard Institute (CLSI) criteria, using the following discs; gentamicin (10 µg), ciprofloxacin (5 µg), ofloxacin (5 µg), amoxicillin-clavulanate (30 µg), cefuroxime (30 µg), ceftriaxone (30 µg) ceftazidime (30 µg), cloxacillin (5 µg), meropenem (10 µg) (all from Abtek Biologicals Ltd, Liverpool, U.K). These were then incubated at 37°C for 18 hrs. Sensitivity pattern was determined by measuring the zones of inhibition with a calibrated ruler and comparing with the control organism. Interpretative criteria for susceptibility tests were according to CLSI^22^.

An oxacillin disc (1 µg) was used to detect methicillin resistance. Sensitivity was read after incubation for 24 hrs at 35° C. Isolates were regarded as sensitive or resistant according to CLSI criteria^23^.

Multiple antibiotic resistance (MAR) index was calculated for each isolate using the formular;

### Chromosomal DNA Extraction

DNA extraction was carried out as previously described^24^; Isolates were harvested into 1.0 ml of sterile water, vortexed to mix, and centrifuged at 10,000 r.p.m for 5 min. The supernatant was discarded and the pellets were washed again with sterile water. After this, 200µl of sterile water was added to the pellets, the pellets were vortexed to homogenize, and boiled in a dry bath at 100°C for 10 minutes. This was followed by vortexing and centrifugation at 12,000 r.p.m for 5 min. The supernatant containing the DNA were transferred to another tube and stored at −20°C. The concentration and purity of the extracted DNA was estimated using a nanodrop spectrophotometer.

### PCR amplification of *mecA* gene.

The mecA gene was amplified using the primer set mecA1( AAAATCGATGGTAAAGGTTGGC) and *mecA*2 (5'AGTTCTGCAGTACCGGATTTTGC3') as described by Del Vechio et al (1995)^25^. PCR was performed in a 20 µl of a reaction mixture containing 1X PCR Buffer (Solis Biodyne), 1.5 mM MgCl_2_, 200 µM of each dNTP (Solis Biodyne), 20 pMol of each primer, 2.5 units of TaqDNA polymerase (Solis Biodyne), 10–200 ng of extracted DNA, and sterile distilled water was used to make up the reaction mixture. Thermal cycling was conducted in an Eppendorf Thermal Cycler Nexus series for an initial denaturation of 95°C for 5 min, followed by 30 consecutive cycles of 95°C for 30 sec; 55°C for 30 sec, and 72°C for 1 min. This was followed by a final extension step of 72°C for 10 min. The amplification product was separated on 1.5% agarose gel electrophoresis, visualized by ethidium bromide staining and photographed under Ultraviolet illumination. 100bp DNA ladder (Solis Biodyne) was used as DNA molecular weight standard. A single band with a molecular weight of 533bp signifies the presence of the mecA gene. *S. aureus* ATCC 43300 served as the positive control strain.

### Calculation of sensitivity and specificity

Calculations of sensitivity were made by dividing the number of strains detected as resistant by the susceptibility test method by the total number of strains that were *mecA* positive. Specificity was calculated by dividing the number of strains detected as susceptible by the test method by the total number of strains that were *mecA* negative.

## Statistical analysis

Statistical analysis was by the Chi (X^2^) square test using INSTAT® software. A p value of < 0.05 was deemed statistically significant.

## Result

A total of 91 clinical isolates of *Staphylococcus* species (S. aureus - 50 (54.9%), CoNS - 41 (45.1%)) were recovered from clinical samples during the period of study. Although the isolation rate of *Staphylococcus* species was higher from males (52.7%), gender was however not a risk factor for *Staphylococcal* infection (X^2^ = 0.2637, P > 0.05). There was no significant association in the occurrence of *Staphylococci* and the isolation sites (X^2^ = 0.5481, P> 0.05).

The isolation rate of *Staphylococcus* species from clinical samples was highest from infected wounds of patients (28.8%) while urine samples showed the least isolation rate (5.4%) as seen in Table 1.

**Table 1:** Distribution of *Staphylococcus* species recovered from clinical samples.

Clinical Sample	No of Samples	No of Culture Positive samples	Distribution in clinical samples	
			*S. aureus*	CoNS
Wound Swab	87	62	13 (15.0)	12 (13.8)
Endocervical swab	35	9	1 (2.9)	3 (8.6)
High vaginal swab	39	13	2 (5.1)	1 (2.6)
Eye swab	22	5	2 (9.1)	2 (9.1)
Ear swab	58	22	6 (10.3)	3 (5.2)
Urethral swab	19	3	2 (10.5)	1 (5.3)
Seminal fluid	36	11	9 (25.0)	1 (2.8)
Blood	65	26	7 (10.8)	10 (15.4)
Urine	223	89	6 (2.7)	6 (2.7)
Aspirates	21	4	1 (4.8)	1 (4.8)
Catheter tip	10	5	1 (6.3)	1 (6.3)

Number in parentheses = value in percentage. CoNS- Coagulase negative *Staphylococci*

The anti-microbial susceptibility pattern showed varying degrees of resistance. The highest level of resistance was toceftazidime (*S. aureus* - 68%, CoNS - 75.6%). The antibiotic with the least resistance was meropenem (*S. aureus*-26%, CoNS- 46.3%). The antibiogram is well detailed in Table 2.

**Table 2:** Antimicrobial susceptibility pattern of *Staphylococcus* species recovered from Clinical samples.

Antibiotics	*S. aureus*		CoNS	
	n = 50		n = 41	
	S	R	S	R
Oxacillin	28 (56.0)	22 (44.0)	22 (53.7)	19 (46.3)
Meropenem	37 (74.0)	13 (26.0)	22 (53.7)	19 (46.3)
Ceftriaxone	27 (54.0)	23 (46.0)	16 (39.0)	25 (61.0)
Cefuroxime	31 (62.0)	19 (3.8)	19 (46.3)	22 (53.7)
Ofloxacin	33 (66.0)	17 (34.0)	19 (46.3)	22 (53.7)
Amoxicillin-clavulanate	30 (60.0)	20 (40.0)	21 (51.2)	20 (48.5)
Cloxacillin	26 (52.0)	24 (48.0)	15 (36.6)	26 (63.4)
Ceftazidime	16 (32.0)	34 (68.0)	10 (24.4)	31 (75.6)
Gentamicin	28 (56.0)	22 (44.0)	19 (46.3)	22 (53.7)
Erythromycin	28 (56.0)	22 (44.0)	15 (36.6)	26 (63.4)

CoNS- Coagulase negative *Staphylococci.* S – Sensitive, R – Resistant. Number in parentheses = value in percentage.

Using phenotypic method (with 1µg oxacillin antibiotic disc), the distribution of MRSA and MRCoNS was 44.0% and 46.3% respectively. Multiple antibiotic resistance (MAR) index was calculated for each of the isolates. It was observed that majority of isolates had MAR indices of above 0.2 (*S. aureus* — 60%, CoNS — 80.5%).

**Figure 1:**
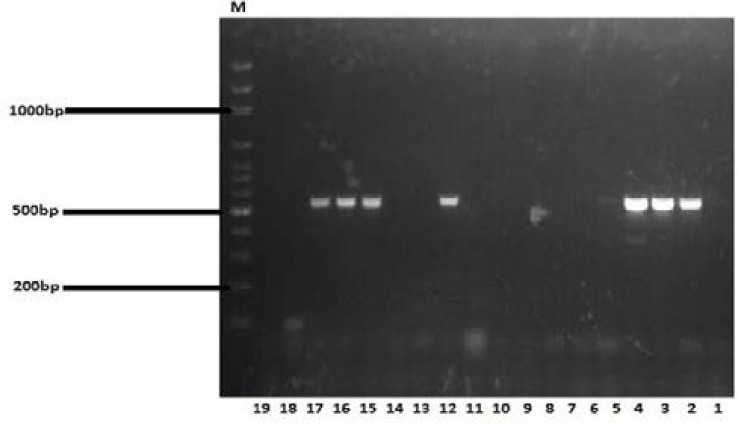
Agarose gel electrophoresis of PCR product amplified from *mecA*genes. M = DNA marker fragments. Lane 1 was the negative control, Lane 2 was the MRSA positive control; *S. aureus* ATCC 43300, Lane 3, 4, 12, 15, 16, and 17 indicate the *mecA* positive samples.. The DNA fragments of 533 bp were amplified from *mecA* gene.

PCR was carried out on all *staphylococcal* isolates to detect *mecA* gene, a gene that confers resistance to methicillin and most β-lactam antibiotics. Figure 1 shows the Agarose gel electrophoresis of PCR product amplified from mecA genes for *S. aureus* and CoNS.

On the whole, PCR analysis showed that 39.6% of *Staphylococcus* species recovered was *mecA* gene positive with 38.0% of *S. aureus* and 41.5% of the CoNS having *mecA* gene respectively. *mecA* gene was markedly distributed in wound swabs (30.5%) and was absent in high vaginal swabs and catheter tips (Table 4). However, there was no significant association between isolation site and the distribution of *mecA* gene among *S. aureus* (X^2^ = 0.7735, P > 0.05), and CoNS (X^2^ = 0.4259, P > 0.05). Seventeen (17) *mecA*+ *S. aureus* were oxacillin resistant (Sensitivity 89.5%) while 16 *mecA*+ CoNS were oxacilin resistant (Sensitivity 94.1%). Twenty-eight (28) of the 31 *mecA- S. aureus* strains were oxacilin sensitive (Specificity 90.3%) while Nineteen (19) of the 24 *mecA*- CoNS were oxacillin sensitive (specificity 79.1%).

**Table 4:** Distribution of *mecA* gene among *Staphylococcus* species from clinical samples

Clinical Sample	N	*S. aureus* *mecA*^+^	N	CoNS *mecA*^+^
Wound swab	13	4 (30.7)	12	7 (58.3)
Endocervical swab	1	0	3	1 (33.3)
High vaginal swab	2	0	1	0
Eye swab	2	1 (50.0)	2	0
Ear swab	6	3 (50.0)	3	2 (66.6)
Urethral swab	2	1 (50.0)	1	1 (100)
Seminal fluid	9	5 (55.5)	1	0
Blood	7	2 (28.5)	10	4 (40.0)
Urine	6	2 (33.3)	6	1 (16.6)
Aspirates	1	1 (100)	1	1 (100)
Catheter tips	1	0	1	0
Total	50	19 (38.0)	41	17 (41.5)

CoNS- Coagulase Negative *Staphylococci*, N = Number of isolates, value in bracket = number in percentages.

There was no association between gender and the distribution of mecA gene among the *Staphylococci* (*S. aureus*; X^2^ = 0.3253, CoNS; X2 = 0.4205; P > 0.05). Similarly, *mecA* gene did not show significant association for the any one of the *Staphylococci* (X^2^ = 0.9604; P >0.05)

## Discussion

In this study, 28.8% of *Staphylococci* isolated were from wound swabs. In a study which had *Staphylococcal* isolates recovered from eight hospitals which cut across South-Western, North-central and North-Eastern Nigeria, more than 80% of the total number of *S. aureus* isolates recovered was from infected wounds^13^. It has been observed that the skin of 80–90% of people is colonized with *S. epidermidis* and that most CoNS infections are acquired from patients own flora^26^. The CoNS have also shown a higher frequency of isolation from wound swabs when compared to other samples^18^. In Nigeria, CoNS is one of the common causes of infections of open fractures in wounds and delay in wound debridement has been reported to be a major predisposing factor to wound infection^28^. Similarly, *S. aureus* is found on the skin, axilla, anterior nares and groins as normal flora^27^. It is therefore not hard to imagine that it would be implicated in wound infection as there is a breach in the structural integrity of the skin.

Resistance was observed to oxacillin in this study, 44.0% and 46.3% for *S. aureus* and the CoNS respectively. This is in contrast to an Iranian study, where resistance was 88% and 60.3% for *S. aureus* and CoNS respectively^29^. Oxacillin and methicilin are hardly used in our setting as compared with the Iranian study which noted the rampant use of oxacillin. Our report is however similar to observations by Olowe et al in Osogbo, Nigeria, where 40.4% of *S. aureus* clinical isolates were resistant to oxacillin. Similarly, demonstrable levels of resistance was observed for meropenem (*S. aureus*- 26%, CoNS- 46.3%), though the drug is expensive and one of last resort in our locality^30^.

The study also shows that majority of *staphylococcal* isolates were multidrug resistant, with MAR indices > 0.2 (*S. aureus* — 60%, CoNS — 80.5%). Worthy of note is the high prevalence of multidrug resistant CoNS. The finding is similar to an observation by Akinjogunla and Enabulele (2010). However, in their study on *Staphylococcal* isolates from ear swabs of patients with otitis media, they found that 19.2% of *S. aureus* and 9.2% of CoNS had MAR indices above 0.8^31^. An MAR index higher than 0.2 has been said to be indicative of isolates originating from an environment where antibiotics were often used^32^, the high indexes from this study therefore give credence to the observation of many researchers in Nigeria on the prevailing practice of indiscriminate use of antibiotics and lack of Laboratory guidance before institution of antimicrobial therapy^16^,^17^, as they may have induced resistance mechanisms over time in these versatile opportunists.

Using PCR method, 38.0% of *S. aureus* isolates and 41.5% of the CoNS had mecA gene respectively. Using phenotypic method (with oxacillin antibiotic disc) however, the distribution of MRSA and MRCoNS was 44.0% and 46.3% respectively. Differing rates in susceptibility pattern has been observed by researchers. Olowe et al in drawing comparisons between methicillin, oxacillin, cefoxitin and the gold standard PCR, observed that all the antibiotics mentioned slightly overestimated methicillin resistance in *S. aureus*^30^. Hyperproduction of β-lactamase enzyme has been observed among mecA negative MRSAs in Nigeria^35^. Besides the challenge of heterogeneity of the strains, this could be a resistance mechanism for oxacillin-resistant *mecA* gene negative *Staphylococci* as observed in this study, more research is therefore needed. There was no significant association between the distribution of *Staphylococci* and the distribution of mecA gene among clinical samples in this study (P > 0.05), as all samples showed some percentage distribution of the gene with the exception of catheter tips and high vaginal swabs. *mecA* gene was markedly distributed among the *staphylococci* recovered from wound swabs in this study (*S. aureus*-30.7%, CoNS-58.3%). This is comparable to a study in Osogbo (*S. aureus*- 52.2%)^30^, and two multi-centre studies evaluating the prevalence of *mecA* gene among the *staphylococci* from clinical samples^13^,^17^.

Noteworthy in this study is the rising prevalence of MRSA and MRCoNS from blood of patients showing signs of septicaemia, as 28.5% of *S. aureus* and 40.0% of the CoNS were *mecA* positive. This is in sharp contrast to some studies in Nigeria in which all *S. aureus* isolated from the blood of patients showing similar symptoms were *mecA* gene negative^17^,^30^, however, the gene was detected among CoNS notably *S. haemolyticusca* having septicemia in Lagos, Nigeria^13^. Some researchers have observed the rising prevalence of MRCoNS from patients with septicaemia in India; 12% and 54% respectively^33^, ^34^.

In our country, little attention has been accorded the CoNS to the extent of screening for *mecA* gene among them. Literature is however growing on its pathogenicity and increased isolation from clinical infections^18^,^19^. The marked distribution of *mecA* gene among these isolates (41.5%), as well as the insignificant difference in their isolation rate and distribution of *mecA* gene when compared with *S. aureus* recovered from clinical samples is a pointer to the fact that they should be accorded equal status as opportunistic pathogens (P > 0.05).

## Conclusion

Summarily, this study notes a rising percentage in the distribution of *mecA* gene among the *Staphylococci* recovered from clinical samples in Benin when compared with previous studies in our country, it reports increasing level of multidrug resistant *staphylococci* with high MAR index, it reiterates the continued practice of indiscriminate use of antibiotics and emphasizes laboratory guidance before institution of anti-microbial therapy. Efforts should also be made to enact regulations on antibiotic usage. Periodic review of susceptibility pattern, molecular epidemiological surveys and surveillance is equally imperative. These when implemented promises to stem the tide of anti-mi crobial resistance.

## Figures and Tables

**Table 3:** Multiple antibiotic resistance (MAR) index of *Staphylococcus* species recovered from clinical samples.

MAR Index	*S. aureus*	CoNS
0.2	5 (16.0)	1(2.8)
0.3	2 (4.0)	3 (8.3)
0.4	1 (2.0)	1 (2.8)
0.5	6 (12.0)	5 (13.8)
0.6	1 (2.0)	1(2.8)
0.7	2 (4.0)	4 (11.1)
0.8	5 (10.0)	3 (8.3)
0.9	8 (16.0)	7 (19.4)
1.0	7 (14.0)	8 (22.2)

Number in parentheses = value in percentage.

CoNS- Coagulase negative *Staphylococci*
